# Evidence of a Causal Link Between the Well-Being Spectrum and the Risk of Myocardial Infarction: A Mendelian Randomization Study

**DOI:** 10.3389/fgene.2022.842223

**Published:** 2022-04-28

**Authors:** Gull Rukh, Shafqat Ahmad, Lars Lind, Helgi Birgir Schiöth

**Affiliations:** ^1^ Functional Pharmacology and Neuroscience, Department of Surgical Sciences, Uppsala University, Uppsala, Sweden; ^2^ Molecular Epidemiology, Department of Medical Sciences, Uppsala University, Uppsala, Sweden; ^3^ Preventive Medicine Division, Harvard Medical School, Brigham and Women’s Hospital, Boston, MA, United States; ^4^ Cardiovascular Epidemiology, Department of Medical Sciences, Uppsala University, Uppsala, Sweden

**Keywords:** wellbeing, cardiovascular disease, Mendelian randomization, depressive symptoms, neuroticism, positive affect, life satisfaction

## Abstract

Epidemiological studies have provided extensive evidence regarding the role of psychological risk factors in the pathogenesis of cardiovascular disease (CVD), but whether these associations are causal in nature is still unknown. We aimed to investigate whether the association between the wellbeing spectrum (WBS; derived from four psychological traits including life satisfaction, positive affect, neuroticism, and depressive symptoms) and CVD risk is causal. By employing a two-sample Mendelian randomization (MR) approach, the effect of the WBS on four CVD outcomes, including atrial fibrillation, heart failure, myocardial infarction, and ischemic stroke, was investigated. The genetically predicted WBS was associated with 38% lower risk for heart failure (odds ratio (OR): 0.62; 95% confidence interval [CI]: 0.50–0.78; *P*: 2.2 × 10^−5^) and 40% reduced risk of myocardial infarction (OR: 0.60; 95% CI: 0.47–0.78; *P*: 1.1 × 10^−4^). Of the WBS constituent traits, only depressive symptoms showed a positive causal association with heart failure and myocardial infarction. Neither WBS nor WBS constituent traits were associated with atrial fibrillation and ischemic stroke. In multivariable MR analyses, when genetic instruments for traditional CVD risk factors were also taken into consideration, the WBS was causally associated with a reduced risk for heart failure (OR: 0.72; 95% CI: 0.58–0.88; *P*: 0.001) and myocardial infarction (OR: 0.67; 95% CI: 0.52–0.86; *P*: 0.002). This study provides evidence that a higher WBS is causally associated with a decreased risk of developing CVD and, more specifically, myocardial infarction; moreover, the association is mainly driven by depressive symptoms. These results support current guidelines that suggest improving psychological wellbeing may help in reducing the burden of cardiovascular disease.

## 1 Introduction

Wellbeing is a broad and complex construct that consists of many aspects and cannot be fully represented by any one measure (Tov et al., 2018). In the current literature, well-being is characterized by two distinct theories: subjective or hedonic wellbeing defined as pleasure attainment and life satisfaction (Kahneman and Schwarz, 1999) and psychological/eudaimonic wellbeing defined as the fulfillment of human potential (Ryan and Deci, 2001). Studies evaluating the relationship between subjective and psychological wellbeing revealed that both measures are highly correlated yet distinguishable constructs, emphasizing the inclusion of both types to yield a more integrated conceptualization of wellbeing (Kashdan et al., 2008; Baselmans and Bartels, 2018).

Evidence from observational studies suggests that both positive and negative psychological factors have an effect on cardiovascular health. Positive psychological wellbeing has been linked with better cardiovascular health, lower incidence of cardiovascular disease (CVD) in healthy populations, and lower risk of adverse outcomes among cardiac patients (Sin, 2016). Moreover, negative psychological factors have been associated with the development and progression of the CVD risk (Chida and Steptoe, 2009; Roest et al., 2010; Whooley and Wong, 2013). However, it is difficult to draw causal inferences based on observational studies due to the likelihood of reverse causation and residual confounding (Lawlor et al., 2008).

Mendelian randomization (MR) is a method that is used to infer causality between an exposure and an outcome by using genetic variants as instrumental variables (Lawlor et al., 2008). Among psychological factors, depression is considered an important public health issue. Recently, many MR studies have observed causal associations between depression and various health outcomes including type 2 diabetes (T2D) and CVD (Khandaker et al., 2020; Mulugeta et al., 2020; Tang et al., 2020). Other than depression, there have been only few MR studies that investigated causal associations between individual psychological factors or wellbeing and health outcomes. One such MR analysis, relying on only a single-item measure of happiness, observed no clear evidence of a causal effect between subjective wellbeing and cardiometabolic traits (Wootton et al., 2018). However, the genetic instrument was composed of only three genome-wide significant SNPs, predicting subjective wellbeing from the prior literature study (Okbay et al., 2016), and only one SNP was replicated in the United Kingdom Biobank data. Therefore, a more liberal *p*-value threshold of *p* < 5 × 10^−5^ was used to increase the number of SNPs available for the instrument, resulting in incorporating a large number of irrelevant SNPs, but the selected genetic variants still explained less than 0.01% of the variance in subjective wellbeing.

Recently, Baselmans *et al.* conducted a multivariate genome-wide meta-analysis of wellbeing related traits (i.e., life satisfaction, positive affect, neuroticism, and depressive symptoms) collectively referred to as the wellbeing spectrum (WBS) (Baselmans et al., 2019) and identified 231 independent genetic variants in association with the WBS by considering a unitary effect of SNPs on all of the four WBS constituent traits (Baselmans et al., 2019). High genotypic and phenotypic correlations between these traits strongly suggest a common underlying biology.

In the current study, our primary aim was to investigate the association between the WBS (as a composite trait) and four CVD outcomes, including atrial fibrillation, myocardial infarction, heart failure, and ischemic stroke, by employing a two-sample MR approach. Our secondary aim was to investigate the causal association between WBS and CVD outcomes independent of traditional CVD risk factors (body mass index (BMI), high-density lipoprotein cholesterol (HDL), low-density lipoprotein cholesterol (LDL), systolic blood pressure (SBP), smoking, and T2D). Moreover, we aimed to investigate the causal association between each of the four WBS traits (life satisfaction, positive affect, neuroticism, and depressive symptoms) and CVD outcomes.

## 2 Methods

### 2.1 Data Sources

Summary data for the genetic variants associated with the WBS and traits such as life satisfaction, positive affect, neuroticism, and depressive symptoms were extracted from the previously published genome-wide association study (GWAS) by Baselmans et al. (2019). For each of the CVD risk factor traits (BMI, HDL, LDL, SBP, smoking, and T2D), genetic variants were identified using previously published corresponding GWAS (Willer et al., 2013; Locke et al., 2015; Evangelou et al., 2018; Xue et al., 2018; Karlsson Linnér et al., 2019) and extracted from United Kingdom Biobank imputed genotype data (Biobank UK, 2018). In the United Kingdom Biobank study, all participants provided written informed consent, and ethical approval was granted by the North West Multi-Centre Research Ethics Committee. All of the published GWASs have existing ethical permission from their respective institutional review boards and have included informed consent from participants. However, as the present study was derived from summary-level data, additional ethics approval was not required for this study.

The corresponding data for genetic associations with CVD outcome traits were obtained from the following consortia: the Atrial Fibrillation (AFGen) consortium with data on atrial fibrillation (Christophersen et al., 2017), the Heart Failure Molecular Epidemiology for Therapeutic Targets (HERMES) consortium with data related to heart failure (Shah et al., 2020), the MEGASTROKE consortium with data about ischemic stroke (Malik et al., 2018), and the Coronary Artery Disease Genome-wide Replication and Meta-analyses (CARDIoGRAM) plus The Coronary Artery Disease (C4D) Genetics (CARDIoGRAMplusC4D) consortium with data on myocardial infarction (Nikpay et al., 2015). For each trait, studies having the largest sample size from populations of similar ancestry with the minimum sample overlap were selected. The percentage of sample overlap is provided in Supplementary Table S1. Since there is a substantial sample overlap between WBS and two of the CVD outcome traits (atrial fibrillation and heart failure), the findings related to both of these CVD outcomes should be interpreted with caution. The GWAS study was used to identify genetic variants corresponding to each trait. The sample size used and the dataset source are provided in Table 1.

**TABLE 1:** Description of GWAS studies used for each trait.

Trait	First author (year)	Sample size	Ancestry	PMID	Web source
Exposures					
Wellbeing spectrum^a^	Baselmans BML (2019)	2,370,390	European	30643256	https://www.ebi.ac.uk/gwas/publications/30643256
Positive affect	Baselmans BML (2019)	410,603	European	30643256	https://www.ebi.ac.uk/gwas/publications/30643256
Life satisfaction	Baselmans BML (2019)	80,852	European	30643256	https://www.ebi.ac.uk/gwas/publications/30643256
Neuroticism	Baselmans BML (2019)	582,989	European	30643256	https://www.ebi.ac.uk/gwas/publications/30643256
Depressive symptoms	Baselmans BML (2019)	1,295,946	European	30643256	https://www.ebi.ac.uk/gwas/publications/30643256
CVD outcomes					
Atrial fibrillation	Christophersen IE (2017)	17,931 cases; 115,142 controls	European (89%)	29892015	http://www.broadcvdi.org/
Heart failure	Shah S (2020)	47,309 cases; 930,014 controls	European	31919418	https://www.hermesconsortium.org/
Myocardial infarction	Nikpay M (2015)	42,592 cases; 123,814 controls	European (77%)	26343387	http://cardiogramplusc4d.org/
Ischemic stroke	Malik R (2018)	40,585 cases; 406,111 controls	European	29531354	https://www.megastroke.org
Cardiovascular risk factors^b^					
BMI	Locke AE (2015)	354,831	European	25673413	https://www.dropbox.com/s/5rvvv82uomug5ah/23104_raw.gwas.imputed_v3.both_sexes.tsv.bgz?dl=0 -O 23104_raw.gwas.imputed_v3.both_sexes.tsv.bgz
HDL	Willer CJ (2013)	315,133	European	24097068	https://www.dropbox.com/s/sn30890f64p0htu/30760_raw.gwas.imputed_v3.both_sexes.tsv.bgz?dl=0 -O 30760_raw.gwas.imputed_v3.both_sexes.tsv.bgz
LDL	Willer CJ (2013)	343,621	European	24097068	https://www.dropbox.com/s/2msvdv4axfz362b/30780_raw.gwas.imputed_v3.both_sexes.tsv.bgz?dl=0 -O 30780_raw.gwas.imputed_v3.both_sexes.tsv.bgz
Smoking (non-smoker)	Karlsson LR (2019)	359,706	European	30643258	https://www.dropbox.com/s/f60w61ifky3wkwh/20116_0.gwas.imputed_v3.both_sexes.tsv.bgz?dl=0 -O 20116_0.gwas.imputed_v3.both_sexes.tsv.bgz
Systolic blood pressure	Evangelou E (2018)	340,159	European	30224653	https://www.dropbox.com/s/cs6sm1a8wnle966/4080_raw.gwas.imputed_v3.both_sexes.tsv.bgz?dl=0 -O 4080_raw.gwas.imputed_v3.both_sexes.tsv.bgz
Type 2 diabetes	Xue A (2018)	360,192	European	30054458	https://www.dropbox.com/s/en89arzlpienvlj/E4_DM2.gwas.imputed_v3.both_sexes.tsv.bgz?dl=0 -O E4_DM2.gwas.imputed_v3.both_sexes.tsv.bgz

a Primary exposure.

b Summary data were obtained from United Kingdom Biobank, Neale Lab and first author, and PMID refer to the original study from which the genetic variants were identified; HDL: high-density lipoprotein cholesterol; LDL: low-density lipoprotein cholesterol; SNPs: single nucleotide polymorphisms.

### 2.2 Identification of Genetic Instruments

For each trait, instrumental variables were constructed from SNPs showing a GWAS significant association (P < 5 × 10^−8^) with the respective trait. The instrumental variable for the WBS included 231 independent SNPs identified by Baselmans et al. (2019) *via* a two-stage approach. In the first stage, they conducted univariate genome-wide meta-analyses for each of the four traits including life satisfaction, positive affect, neuroticism, and depressive symptoms. In the second stage, the resulting four datasets were meta-analyzed (multivariate genome-wide association meta-analyses) into the WBS. For the purpose of multivariate genome-wide association meta-analyses, the estimated SNP effects on neuroticism and depressive symptoms were reversed to ensure a positive correlation with life satisfaction and positive affect (Baselmans et al., 2019). We further used LDlink (version 3.8) (Machiela and Chanock, 2015) to assess the linkage disequilibrium (LD; defined as *r* ^2^ > 0.1) between the SNPs and found 22 SNP pairs in LD with each other. The SNP within each correlated pair that had the weakest association with wellbeing was excluded, leaving 209 independent SNPs for the WBS for further consideration. Additionally, any palindromic SNPs that have a minor allele frequency above 0.42 were excluded to ensure that the effects of the SNPs on exposure correspond to the same allele as their effects on outcomes (Davey Smith and Hemani, 2014). We also conducted univariable MR analyses to investigate the causal association of each of the four WBS traits (life satisfaction, positive affect, neuroticism, and depressive symptoms) and CVD risk factor traits with CVD outcome traits. Instrumental variables for each WBS trait were identified from model-averaging genome-wide association meta-analyses by Baselmans et al. (2019). Similar to the WBS, the instrumental variable for each of the WBS traits was assessed for LD, and from each instrument, the SNPs in LD were excluded. Thus, the instruments for life satisfaction, positive affect, neuroticism, and depressive symptoms comprised 126, 155, 209, and 198 associated SNPs, respectively. For all CVD risk factor traits, genetic instruments were identified from the corresponding published GWAS of the predominant European ancestry (Locke et al., 2015; Willer et al., 2013; Evangelou et al., 2018; Xue et al., 2018; Karlsson Linnér et al., 2019), and the summary statistics data were downloaded from the United Kingdom Biobank GWAS conducted by Neale Lab (http://www.nealelab.is/uk-biobank). The identified instruments for BMI, HDL, LDL, smoking, SBP, and T2D comprised 97, 71, 58, 223, 130, and 139 associated SNPs, respectively. For all the identified SNPs, summary statistics for each of the four CVD outcome traits (atrial fibrillation, heart failure, myocardial infarction, and ischemic stroke) were obtained from the largest available GWAS meta-analysis among individuals of the European ancestry (Nikpay et al., 2015; Malik et al., 2018; Roselli et al., 2018; Shah et al., 2020). Effect alleles were harmonized across all the summary data before analyses.

### 2.3 Statistical Analyses

#### 2.3.1 Univariable MR Analyses

Primarily, we investigated the causal association between the WBS and four CVD outcome traits by employing a two-sample MR approach and conducting univariable MR analyses. We used three different methods: 1) the inverse variance weighted method, 2) the weighted median method, and 3) the MR-Egger method. Each of these methods is based on different assumptions about horizontal pleiotropy, and consistent effect estimates across all three methods build confidence in the obtained estimates and are less likely to be false positives (Lawlor et al., 2016). The inverse variance weighted method provides the highest precision, but it is based on the assumption that all genetic variants are valid instrumental variables (Burgess et al., 2017). The weighted median method gives less precise estimates than the inverse variance weighted method but provides reliable estimates if at least 50% of the genetic variants are valid instrumental variables. The MR-Egger method does not make any assumptions about genetic variants and has low precision, but it can detect and correct for pleiotropy and provide a causal estimate even if all genetic variants have pleiotropic effects on the outcome (Burgess et al., 2017). All analyses were conducted using the *TwoSampleMR* package in R (Hemani et al., 2018). All statistical tests were two-sided. A Bonferroni corrected *p* value below 0.0125 (where *α* = 0.05/4 CVD outcome traits) was considered as strong evidence of association, and the *p* values between 0.0125 and 0.05 were considered to be suggestive of association and required further confirmation. Thus, the result of the inverse variance weighted method was considered the primary method of statistical evidence of the causal effect (following Bonferroni correction) if it is directionally consistent with MR-Egger estimates and has no statistical evidence of horizontal pleiotropy (MR-Egger intercept).

#### 2.3.2 Multivariable MR Analyses

We conducted multivariable MR as a secondary analysis to investigate the direct causal effect of the WBS on CVD outcomes independent of traditional CVD risk factors. In multivariable MR, each CVD outcome (atrial fibrillation, heart failure, myocardial infarction, and ischemic stroke) was regressed on genetically predicted values of WBS, BMI, HDL and LDL, smoking, SBP, and T2D, meaning that estimates represent the direct effect of the WBS on CVD outcomes that is not mediated *via* any of the listed risk factors. Of the total 923 SNPs, summary statistics for 807 SNPs were available for the WBS. Summary statistics for CVD risk factors and CVD outcome traits were extracted for these 807 SNPs. Furthermore, palindromic SNPs were excluded when harmonizing exposure and outcome data. The multivariable MR analyses were conducted using the inverse variance weighted method by employing the *mv_multiple*() function in the *TwoSampleMR* package in R. A Bonferroni corrected *p* value below 0.0125 (where *α* = 0.05/4 CVD outcome traits) was considered as strong evidence of direct association, and the *p* values between 0.0125 and 0.05 were considered to be suggestive of association.

### 2.4 Pleiotropy Assessment

MR-Egger intercept and MR-PRESSO (MR-Pleiotropy RESidual Sum and Outlier) methods were employed to test the presence of directional pleiotropy. MR-PRESSO analyses comprised three tests: the MR PRESSO *global test* to detect horizontal pleiotropy, the *outlier-corrected causal estimate* to correct for detected horizontal pleiotropy, and the MR-PRESSO *distortion test* to estimate the significant difference in the causal estimate after adjustment for outliers (at *p* < 0.05). Outlier-corrected causal estimates were used when both global and distortion tests were significant (i.e., *p* < 0.05).

## 3 Results

### 3.1 Univariable MR Analyses of the WBS and CVD Outcomes

**FIGURE 1:**
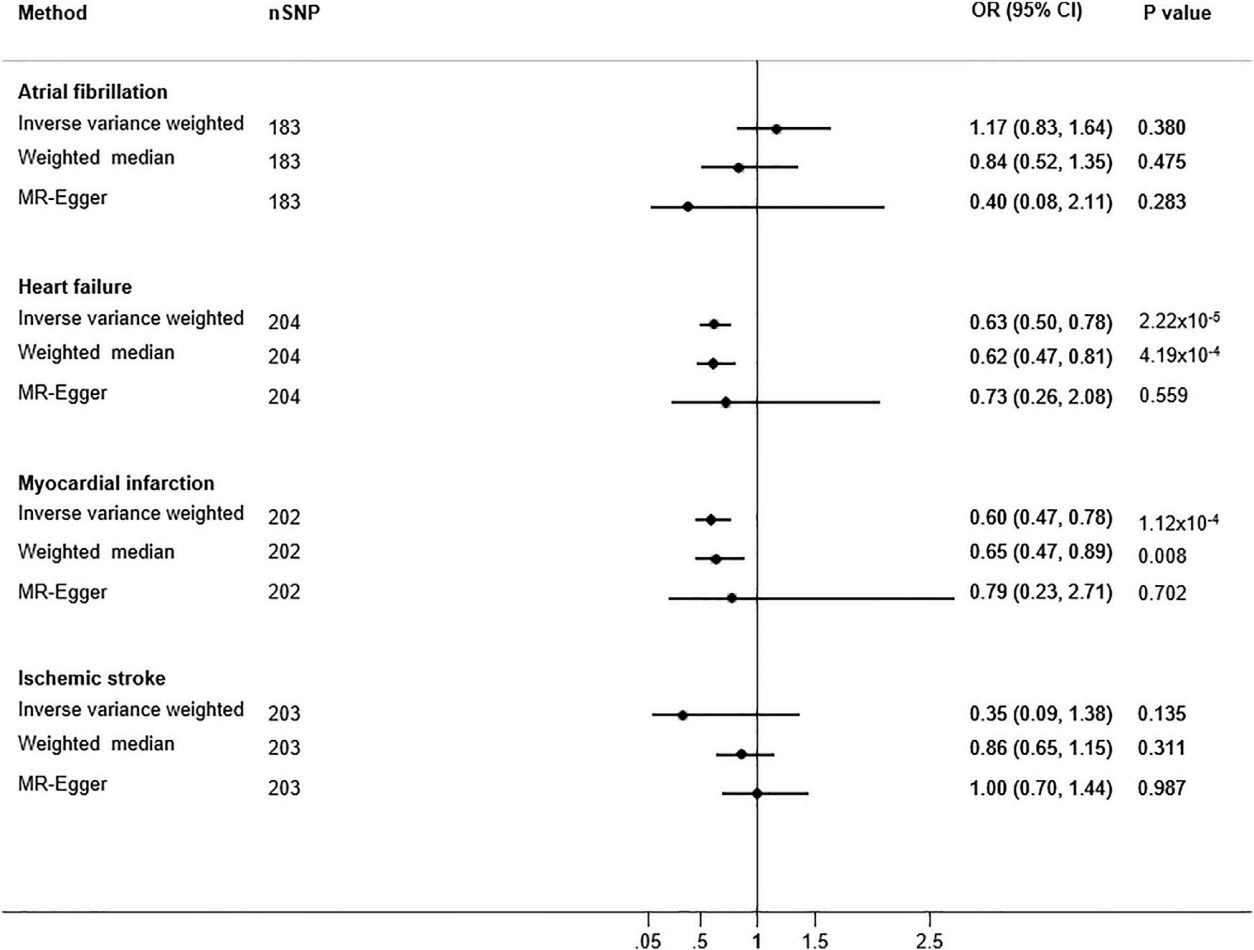
Two-sample univariable Mendelian randomization analyses of the wellbeing spectrum with cardiovascular disease. Odds ratios (ORs) of cardiovascular disease outcomes are for per standard deviation increase in psychological wellbeing. A genetic instrument for the wellbeing spectrum comprised 209 genetic variants. nSNP: number of single nucleotide polymorphisms (the number of SNPs varies due to the unavailability of the SNPs in the outcome summary statistics or not passing the quality control procedures).

The associations between the WBS and CVD outcomes are shown in Figure 1. After Bonferroni correction, the genetically predicted WBS was associated with a lower risk for both heart failure (OR: 0.63; 95% confidence interval [CI]: 0.50–0.78; P: 2.22 × 10^−5^) and myocardial infarction (OR: 0.60; 95% CI: 0.47–0.78; P: 1.12 × 10^−4^) by the inverse variance weighted method. In the sensitivity analyses, results through the weighted median method also provide strong evidence for an inverse association between the WBS and both heart failure (OR: 0.62; 95% CI: 0.48–0.81; P: 4.19 × 10^−4^) and myocardial infarction (OR: 0.65; 95% CI: 0.47–0.89; P: 0.008). However, by using the MR-Egger method, the association results of the WBS with both heart failure (OR: 0.73; 95% CI: 0.26–2.08; P: 0.56) and myocardial infarction (OR: 0.79; 95% CI: 0.23–2.71; P: 0.70) were non-significant but directionally consistent with the results from the inverse variance weighted and weighted median methods, and the intercept did not indicate pleiotropic bias (P^HR^_intercept_ = 0.76 and P^MI^_intercept_ = 0.67). Moreover, the genetically predicted WBS did not associate with atrial fibrillation and ischemic stroke in both main and sensitivity MR analyses (Figure 1). The MR-PRESSO method identified one outlier SNP for heart failure and ischemic stroke and two outlier SNPs for myocardial infarction. Although the global test was significant, the distortion test did not identify significantly different estimates following adjustment for outliers (Supplementary Table S2). No outlier SNPs were identified in the MR-PRESSO analysis for atrial fibrillation. Cochran’s Q indicated the presence of heterogeneity (Supplementary Table S3), but the MR-Egger intercept showed no evidence of directional pleiotropy (Supplementary Table S2).

### 3.2 Univariable MR Analyses of WBS Constituent Traits and CVD Outcomes

The association of four WBS traits (life satisfaction, positive affect, depressive feelings, and neuroticism) with CVD outcomes was also investigated. The MR-Egger intercept showed no evidence for directional pleiotropy (Supplementary Table S2). Cochran’s Q for the presence of heterogeneity between the WBS and CVD outcomes is shown in Supplementary Table S3.

The associations between life satisfaction and incident CVD traits are shown in Supplementary Figure S1. In the main analyses (inverse variance weighted method), after Bonferroni correction, genetically predicted life satisfaction was associated with a lower risk of heart failure (OR: 0.76; 95% CI: 0.63–0.92; *P*: 0.005). In the sensitivity analyses, the weighted median method provided only suggestive evidence of an association between life satisfaction and heart failure (OR: 0.76; 95% CI: 0.59–0.98; P: 0.037) whereas the MR-Egger method suggested a null association and was not directionally consistent with inverse variance and weighted median methods. No associations were observed for life satisfaction with atrial fibrillation, myocardial infarction, and ischemic stroke (Supplementary Figure S1). No significant outlier SNPs were identified by MR-PRESSO analyses, and the MR-Egger intercept showed no evidence for directional pleiotropy (Supplementary Table S2).

The associations between positive affect and CVD outcomes are shown in Supplementary Figure S2. No evidence for associations was observed for positive affect with any of the studied CVD traits by the inverse variance weighted method, except heart failure (OR: 0.76; 95% CI: 0.64–0.92; P: 0.003). In the sensitivity analyses, no significant associations were observed between genetically predicted positive affect and any of the CVD traits using weighted median and MR-Egger methods. MR-PRESSO analyses identified one outlier SNP for myocardial infarction, but the distortion test did not identify significantly different estimates, following adjustment for outliers (Supplementary Table S2).

The associations between neuroticism and CVD outcomes are shown in Supplementary Figure S3. Genetically predicted neuroticism was associated with a higher risk for both heart failure (OR: 1.24; 95% CI: 1.09–1.42; P: 0.002) and myocardial infarction (OR: 1.24; 95% CI: 1.06–1.46; P: 0.008) by the inverse variance weighted method. In the sensitivity analyses, results obtained by the weighted median method revealed consistent estimates but of low precision, but the MR-Egger method suggested a null association. In addition, the estimates obtained by the MR-Egger method were not directionally consistent with inverse variance weighted and weighted median methods. No associations were observed for neuroticism with atrial fibrillation and ischemic stroke. The MR-PRESSO method identified one outlier SNP for heart failure and two outlier SNPs for myocardial infarction. Although the global test was significant, the distortion test did not identify significantly different estimates following adjustment for outliers (Supplementary Table S2).

The results for the association between depressive symptoms and CVD outcomes are shown in Supplementary Figure S4. Genetically predicted depressive symptoms were associated with a higher risk for both heart failure (OR: 1.48; 95% CI: 1.21–1.79; P: 9.6 × 10^−5^) and myocardial infarction (OR: 1.53; 95% CI: 1.20–1.96; P: 6.2 × 10^−4^) by the inverse variance weighted method. Similar associations were observed by the weighted median method. However, by the MR-Egger method, the association results of depressive symptoms with both heart failure and myocardial infarction are non-significant but directionally consistent with the results from the inverse variance weighted method, and the intercept did not indicate pleiotropic bias (Supplementary Table S2). No associations were observed between depressive symptoms and atrial fibrillation or ischemic stroke. The MR-PRESSO method identified one outlier SNP each for heart failure and ischemic stroke and two outlier SNPs for myocardial infarction. Although the global test was significant, the distortion test did not identify significantly different estimates, following adjustment for outliers (Supplementary Table S2).

### 3.3 Univariable MR Analyses of CVD Risk Factor Traits and CVD Outcomes

The association of CVD risk factors with CVD outcomes was also investigated. The associations between CVD risk factors and atrial fibrillation are shown in Supplementary Table S4. In the main analyses (inverse variance weighted method), after Bonferroni correction, genetically predicted BMI was associated with a higher risk of atrial fibrillation (OR: 1.08; 95% CI: 1.04–1.11; P: 8.6 × 10^−6^). In the sensitivity analyses, the weighted median method showed similar associations (OR: 1.07; 95% CI: 1.02–1.12; P: 0.005) whereas the MR-Egger method suggested a null association, but estimates were directionally consistent with the other two MR methods. No associations were observed between the rest of the CVD risk factors and atrial fibrillation (Supplementary Table S4).

The associations between CVD risk factors and heart failure are shown in Supplementary Table S5. All CVD risk factors were associated with heart failure by the inverse variance weighted method (*p* < 0.002 for all). Similar estimates were observed by the weighted median and MR-Egger methods for all traits except T2D, where both sensitivity tests showed null associations.

The associations between CVD risk factors and myocardial infarction are shown in Supplementary Table S6. All CVD risk factors were significantly associated with myocardial infarction by the inverse variance weighted method (*p* < 0.0003 for all). In the sensitivity analyses, results obtained by the weighted median method revealed consistent estimates, and the estimates obtained by the MR-Egger method were directionally consistent with both MR methods.

The results for the association between CVD risk factors and ischemic stroke are shown in Supplementary Table S7. Genetically predicted SBP and T2D were associated with an increased risk for ischemic stroke (*p* < 0.0001 for both) by the inverse variance weighted method. Similar associations were observed by the weighted median method and were directionally consistent with the MR-Egger method.

### 3.4 Multivariable MR Analyses of WBS and CVD Outcomes

The multivariable MR inverse variance weighted method was used to obtain direct causal estimates for the association between the WBS and CVD outcomes independent of the traditional CVD risk factors. Results from the multivariable MR analyses suggested an inverse causal association between the WBS and both heart failure (OR: 0.72; 95% CI: 0.58–0.88; P: 0.001) and myocardial infarction (OR: 0.67; 95% CI: 0.52–0.86; P: 0.002) independent of CVD risk factors (Table 2). No significant associations were observed between the WBS and atrial fibrillation or ischemic stroke after adjustment with CVD risk factors.

**TABLE 2:** Multivariable Mendelian randomization analyses for the direct association of the wellbeing spectrum with cardiovascular outcomes after adjusting for traditional cardiovascular risk factors.

Outcome	Exposure	N SNPs	OR	LCI	UCI	P
Atrial fibrillation	**Wellbeing spectrum**	160	1.111	0.794	1.553	0.539
	Body mass index	101	1.064	1.036	1.093	4.63 × 10^−6^
	High-density lipoprotein cholesterol	80	0.845	0.685	1.043	0.117
	Low-density lipoprotein cholesterol	48	0.904	0.806	1.014	0.085
	Systolic blood pressure	29	1.016	1.006	1.027	0.002
	Smoking (never)	73	1.247	0.890	1.747	0.199
	Type 2 diabetes	37	0.553	0.233	1.313	0.179
Heart failure	**Wellbeing spectrum**	178	0.716	0.583	0.879	0.001
	Body mass index	103	1.088	1.070	1.107	1.37 × 10^−22^
	High-density lipoprotein cholesterol	86	0.874	0.767	0.995	0.043
	Low-density lipoprotein cholesterol	49	1.174	1.092	1.262	1.31 × 10^−5^
	Systolic blood pressure	34	1.018	1.012	1.025	3.75 × 10^−8^
	Smoking (never)	79	0.702	0.572	0.861	0.001
	Type 2 diabetes	40	2.851	1.652	4.922	1.69 × 10^−4^
Myocardial infarction	**Wellbeing spectrum**	177	0.666	0.517	0.857	0.002
	Body mass index	102	1.054	1.033	1.077	5.85 × 10^−7^
	High-density lipoprotein cholesterol	86	0.657	0.560	0.771	2.60 × 10^−7^
	Low-density lipoprotein cholesterol	49	1.937	1.767	2.124	4.95 × 10^−45^
	Systolic blood pressure	33	1.030	1.022	1.038	3.45 × 10^−13^
	Smoking (never)	77	0.680	0.526	0.879	0.003
	Type 2 diabetes	40	18.653	9.564	36.378	9.05 × 10^−18^
Ischemic stroke	**Wellbeing spectrum**	178	0.940	0.731	1.210	0.632
	Body mass index	102	1.015	0.994	1.036	0.159
	High-density lipoprotein cholesterol	86	0.924	0.787	1.086	0.338
	Low-density lipoprotein cholesterol	49	1.090	0.997	1.193	0.058
	Systolic blood pressure	34	1.026	1.018	1.034	2.29 × 10^−10^
	Smoking (never)	79	0.766	0.595	0.985	0.037
	Type 2 diabetes	40	5.134	2.611	10.095	2.13 × 10^−6^

OR: odds ratio; LCI: lower 95% confidence interval; UCI: upper 95% confidence interval.; N SNPs: number of single nucleotide polymorphisms (number of SNPs differ due to unavailability in the outcome summary statistics data or not passing quality control procedures).

### 3.5 Reverse MR

Reverse MR analyses were also conducted to investigate whether myocardial infarction has a causal effect on the WBS or on any of the four constituent traits. No significant associations were observed between myocardial infarction and any of the outcome traits (WBS: positive affect, life satisfaction, neuroticism, and depressive symptoms) by all three MR methods. Results for reverse MR analyses are provided in Supplementary Table S8.

## 4 Discussion

**FIGURE 2:**
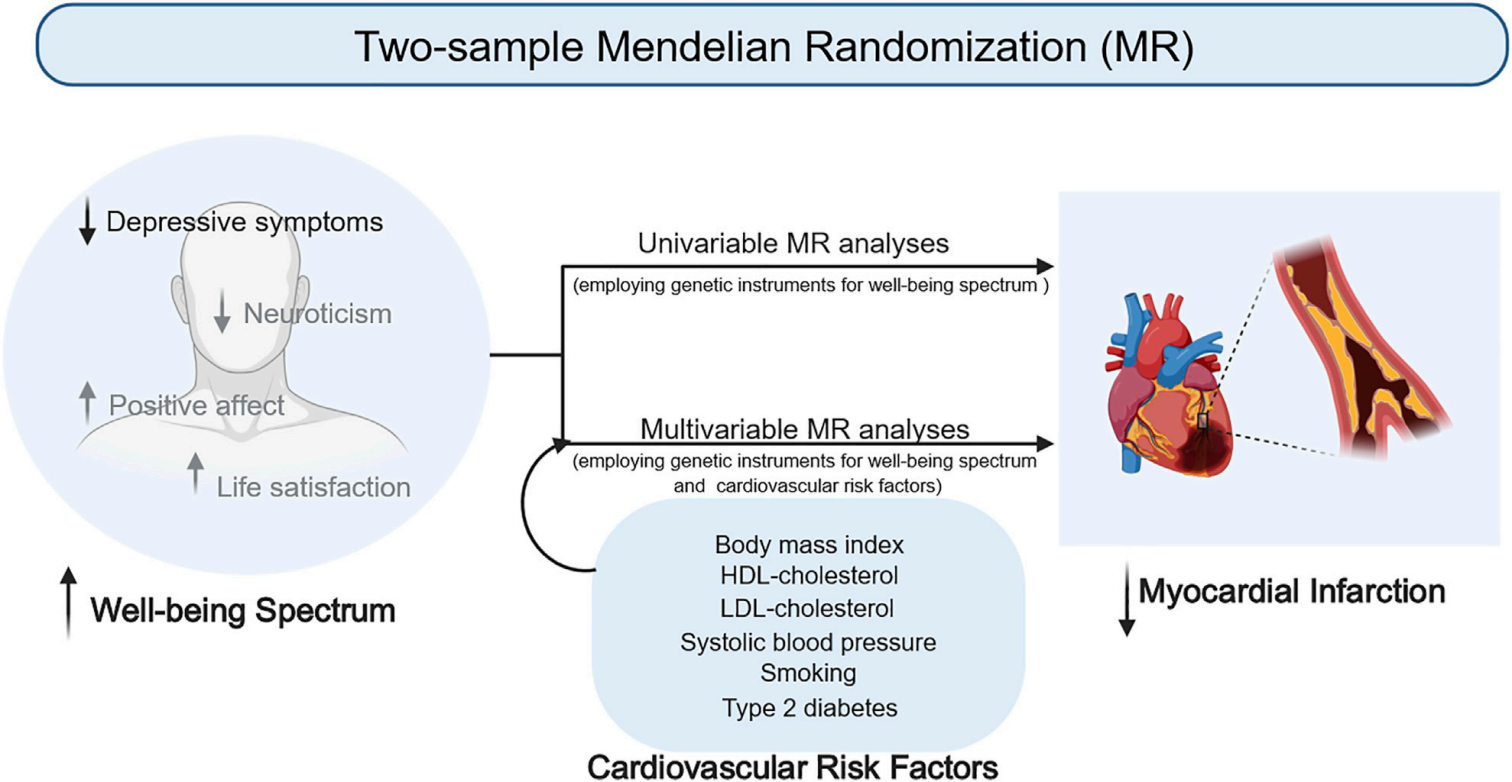
Causal association between the wellbeing spectrum and risk of cardiovascular disease.

This MR study provides clear evidence that a higher WBS may be causally associated with a decreased risk of heart failure and myocardial infarction. While the causal association between the WBS and heart failure needs further confirmation, higher WBS is causally associated with a lower risk of myocardial infarction independent of CVD risk factors (Figure 2). Among the four psychological traits that constitute the WBS, depressive symptoms appear to be the key factors responsible for the causal effect of the WBS on CVD.

Observational studies have focused separately on both negative and positive psychological factors, where negative factors seem to increase and positive factors seem to decrease the risk of CVD (Chida and Steptoe, 2009; Roest et al., 2010; Whooley and Wong, 2013; Sin, 2016). However, it is difficult to draw causal inference from observational studies owing to the issues of reverse causation and residual confounding (Lawlor et al., 2008). In order to overcome these issues, we employed the MR approach to investigate the causality. So far, only one study by Wootton and colleagues has used the MR approach to investigate the causal association between SWB and cardiometabolic health. This study used a single-item measure of happiness to define SWB and failed to find clear evidence of a causal effect between subjective wellbeing and cardiometabolic traits in either direction (Wootton et al., 2018). Our study, to the best of our knowledge, is the first study that incorporated both negative and positive psychological factors in the WBS and evaluated the risk of CVD associated with it. In contrast to the Wootton *et al.* study, we observed clear evidence that higher WBS [a genetic instrument derived from meta-analyzing life satisfaction, positive affect, neuroticism, and depressive symptoms genome-wide association studies, where estimated SNP effects on neuroticism and depressive symptoms were reversed to ensure a positive correlation between all the four traits (16)] is causally associated with a lower risk of having heart failure and myocardial infarction in the univariable MR analyses and observed directionally consistent estimates across the multiple MR methods, ensuring that the findings are less likely to be false positive. The Wootton *et al.* study (Wootton et al., 2018) did investigate coronary artery disease and myocardial infarction, but other than that, we are not aware of any previous MR analyses of well-being in relation to the risk of other CVD traits such as atrial fibrillation, heart failure, and ischemic stroke.

In order to understand the biological mechanisms underlying the potential causal association between wellbeing and CVD, we conducted multivariable MR analyses of the WBS and CVD traits by additionally adjusting for genetic instruments of BMI, HDL, LDL, SBP, smoking, and T2D. We hypothesized that the WBS may lead to specific biological alterations that mediate its effect on CVD. Multivariable MR analyses provide clear evidence of a causal association between the WBS and myocardial infarction independent of CVD risk factors. This implies that improving one’s psychological wellbeing may help in myocardial infarction risk reduction.

Furthermore, we investigated if all or any of the four meta-analyzed traits constituting WBS were causally associated with CVD. In the case of life satisfaction, positive affect, and neuroticism, either there was no evidence of association or resulting estimates were not directionally consistent across different MR methods. Thus, these findings need to be interpreted with caution and require further investigation. Depressive symptoms, on the other hand, provide strong evidence for a causal association with both heart failure and myocardial infarction. These findings suggest that among the four WBS constituent traits, depressive symptoms are mainly responsible for the observed causal association between the WBS and CVD. In agreement with our findings, depression was strongly associated with myocardial infarction in the large multinational, case-control INTERHEART study (Yusuf et al., 2004). A recent study that investigated the comorbidity between coronary heart disease (CHD) and depression using MR analyses did not observe a significant association and suggested that comorbidity between CHD and depression arises largely from shared environmental factors (Khandaker et al., 2020). However, the study focused on CHD and cardiovascular risk factors as causes of depression but did not perform the analyses in the reverse direction, i.e., this study did not investigate the CHD risk associated with a genetic risk score for depression. Moreover, in agreement with this study, the present study also conducted reverse MR analyses and does not support the causal effect of MI on WBS and constituent traits.

Depression has also been found to be more prevalent among patients with acute myocardial infarction (34). Thus, the association between depression and myocardial infarction cannot be simply explained as a reaction to emotional trauma, following a life-threatening health condition. Several CVD risk factors such as hyperlipidemia, T2D, hypertension, and inflammatory biomarkers are known to be associated with the risk of depression, suggesting a shared mechanism between depression and CHD (Colquhoun et al., 2013). Moreover, depression is known to be associated with physiological changes that negatively influence the cardiovascular system such as nervous system activation, cardiac rhythm disturbances, localized and systemic inflammation, and hypercoagulability (Joynt et al., 2003). Furthermore, higher levels of neuroticism have also been shown to increase the risk of CVD in depressed people (Almas et al., 2017). Thus, appropriate monitoring and treating depression may help in improving the overall well-being, improved quality of life, and potentially improved cardiovascular health.

Our study has several strengths that need to be acknowledged. First, our main exposure, WBS, is well-captured, and instead of relying on a single-item measure, it is a composite of multiple psychological factors (both positive and negative) that are highly correlated both phenotypically and genetically, suggesting a common underlying biology. Second, we were also able to investigate the causal associations between the constituent WBS traits and CVD to identify the psychological factors playing a key role in deriving the causal association. Third, we adopted a two-sample analysis strategy and used publicly available summarized data from published large GWAS. This framework allows efficient identification of risk factors that are suitable targets for clinical intervention (Burgess et al., 2015). Last, we employed a large number of SNPs to constitute the genetic instruments and employed multiple MR methods with different assumptions about pleiotropy to ensure that bias due to pleiotropy is unlikely. There are also certain limitations that we would like to highlight. MR estimates can be biased in the presence of sample overlap, and in the case of atrial fibrillation and heart failure, there is a substantial overlap between the samples used for the WBS genome-wide association study and the genome-wide data for these two CVD outcomes. Thus, the results for atrial fibrillation and heart failure outcomes should be interpreted with caution, and further investigation should be conducted in independent samples. Another important limitation is the generalizability of our findings, i.e., our analyses are based on the data predominant of the European ancestry; thus, due to the difference in the LD structure, the generalizability of our findings might be limited for other ethnic groups. Another limitation is the non-availability of individual-level data to conduct the observational analyses. Moreover, lack of evidence does not mean the absence of association. The lack of evidence in some of our analyses could be a consequence of weak instruments; however, consistent estimates were observed using different MR methods, indicating that observed associations are not likely to be by chance findings. Last, we would like to mention that the term “wellbeing spectrum” is not commonly used in the existing literature or clinical practice to refer to the broad spectrum of psychological health that we have evaluated in the present study.

## 5 Conclusion

In the current study, by using the MR approach, we observed clear evidence for a causal effect of the WBS on CVD and more specifically on myocardial infarction. We also found strong evidence that the effect of the WBS on the risk of CVD is mainly driven by the adverse effect of depressive symptoms on myocardial infarction. Our findings stress upon the need to pay attention to mental and psychological wellbeing because of its downstream consequences on cardiovascular health. Our study suggests and further supports current guidelines that steps should be taken to improve psychological health/wellbeing (e.g., depression) in order to prevent and reduce the risk of cardiovascular events (Piepoli et al., 2016).

## Data Availability

Publicly available datasets were analyzed in this study. These data can be found here: most of the data underlying this article are available from the GWAS catalog (https://www.ebi.ac.uk/gwas/downloads/summary-statistics). The data for CVD outcome traits are available to download from their respective consortia (atrial fibrillation: http://www.broadcvdi.org/; heart failure: https://www.hermesconsortium.org/; myocardial infarction: http://cardiogramplusc4d.org/; ischemic stroke: https://www.megastroke.org). The data for CVD risk factors are available to download from Neale Lab GWAS (http://www.nealelab.is/uk-biobank).
