# Supplementary material for: Evidence of a Causal Link Between the Well-Being Spectrum and the Risk of Myocardial Infarction: A Mendelian Randomization Study
Source: Front Genet. 2022 Apr 28;13:842223. doi: 10.3389/fgene.2022.842223 (PMC9096350; doi:10.3389/fgene.2022.842223)
Supplement: Supplementary file 1 [file Table1.DOCX]

Supplementary Material

**List of Supplementary Figures**

Figure S1. Two sample univariable MR analyses showing the association of life satisfaction with CVD outcomes.

Figure S2. Two sample univariable MR analyses showing the association of positive affect with CVD outcomes.

Figure S3. Two sample univariable MR analyses showing the association of neuroticism with CVD outcomes.

Figure S4. Two sample univariable MR analyses showing the association of depressive symptoms with CVD outcomes.

**List of Supplementary Tables**

Table S1. Percentage sample overlap between the genome wide association studies for well-being spectrum and cardiovascular outcomes.

Table S2. Evaluation of directional pleiotropic effects of the genetic instruments for WBS and constituent traits on cardiovascular outcomes.

Table S3. Cochran’s Q test for the presence of heterogeneity between WBS and constituent traits, and cardiovascular disease outcomes.

Table S4. Univariable Mendelian randomization analyses of cardiovascular risk factors with atrial fibrillation.

Table S5. Univariable Mendelian randomization analyses of cardiovascular risk factors with heart failure.

Table S6. Univariable Mendelian randomization analyses of cardiovascular risk factors with myocardial infarction.

Table S7. Univariable Mendelian randomization analyses of cardiovascular risk factors with ischemic stroke.

Table S8. Reverse Mendelian randomization analyses of myocardial infarction (as exposure) with well-being spectrum and four well-being traits (as outcome).

**Figure S1.** Two sample univariable Mendelian randomization (MR) analyses showing the association of life satisfaction with cardiovascular disease (CVD) outcomes using different MR methods. Odds ratios (ORs) of CVD outcomes are for per unit increase in life satisfaction. nSNP refer to the number of single nucleotide polymorphisms (number of the SNPs vary due to the unavailability of the SNPs in the outcome summary statistics or not passing the quality control procedures. CI: Confidence interval.

**Figure S2.** Two sample univariable Mendelian randomization (MR) analyses showing the association of positive affect with cardiovascular disease (CVD) outcomes using different MR methods. Odds ratios (ORs) of CVD outcomes are for per unit increase in life satisfaction. nSNP refer to the number of single nucleotide polymorphisms (number of the SNPs vary due to the unavailability of the SNPs in the outcome summary statistics or not passing the quality control procedures. CI: Confidence interval.

**Figure S3.** Two sample univariable Mendelian randomization (MR) analyses showing the association of neuroticism with cardiovascular disease (CVD) outcomes using different MR methods. Odds ratios (ORs) of CVD outcomes are for per unit increase in life satisfaction. nSNP refer to the number of single nucleotide polymorphisms (number of the SNPs vary due to the unavailability of the SNPs in the outcome summary statistics or not passing the quality control procedures. CI: Confidence interval.

**Figure S4.** Two sample univariable Mendelian randomization (MR) analyses showing the association of depressive symptoms with cardiovascular disease (CVD) outcomes using different MR methods. Odds ratios (ORs) of CVD outcomes are for per unit increase in life satisfaction. nSNP refer to the number of single nucleotide polymorphisms (number of the SNPs vary due to the unavailability of the SNPs in the outcome summary statistics or not passing the quality control procedures. CI: Confidence interval.

**Table S1.** Percentage sample overlap between the genome wide association studies for well-being spectrum and cardiovascular outcomes.

| **Cardiovascular outcome** | **Author (year)** | **GWAS consortia** | **% sample overlap** |
| --- | --- | --- | --- |
| Atrial fibrillation | Christophersen IE (2017) | AFGen | 26.84% |
| Heart failure | Shah S (2020) | HERMES | 43.51% |
| Myocardial infarction | Nikpay M (2015) | CARDIoGRAMplusC4D | 20.05% |
| Ischemic stroke | Malik R (2018) | MEGASTROKE | 13.99% |

**Table S2.** Evaluation of directional pleiotropic effects of the genetic instruments for WBS and constituent traits on cardiovascular outcomes.

| **Exposure** | **Outcome** | **MR-Egger intercept** | **SE** | **P** | **MR-PRESSO** | | | |
| --- | --- | --- | --- | --- | --- | --- | --- | --- |
|  |  |  |  |  | **Global test** | **P** | **Distortion test** | **P** |
| WBS | Atrial fibrillation | 0.007 | 0.005 | 0.200 | 212.787 | 0.075 | NA | NA |
|  | Heart failure | -0.001 | 0.003 | 0.763 | 275.680 | <0.001 | -4.668 | 0.831 |
|  | Myocardial infarction | -0.002 | 0.004 | 0.670 | 265.753 | 0.002 | -2.222 | 0.923 |
|  | Ischemic stroke | 0.006 | 0.005 | 0.189 | 284.928 | <0.001 | -34.735 | 0.686 |
| Depressive symptoms | Atrial fibrillation | 0.002 | 0.005 | 0.720 | 182.797 | 0.249 | NA | NA |
|  | Heart failure | 0.001 | 0.003 | 0.850 | 225.902 | 0.044 | -5.741 | 0.817 |
|  | Myocardial infarction | 0.002 | 0.004 | 0.680 | 247.298 | 0.005 | -2.665 | 0.922 |
|  | Ischemic stroke | 0.002 | 0.004 | 0.666 | 253.304 | 0.002 | -52.894 | 0.609 |
| Neuroticism | Atrial fibrillation | 0.001 | 0.005 | 0.820 | 207.534 | 0.087 | NA | NA |
|  | Heart failure | -0.003 | 0.003 | 0.359 | 294.257 | <0.001 | -6.304 | 0.838 |
|  | Myocardial infarction | -0.003 | 0.004 | 0.431 | 285.289 | <0.001 | -3.399 | 0.923 |
|  | Ischemic stroke | 0.001 | 0.004 | 0.737 | 264.823 | 0.003 | NA | NA |
| Life satisfaction | Atrial fibrillation | 0.007 | 0.007 | 0.357 | 119.390 | 0.213 | NA | NA |
|  | Heart failure | -0.003 | 0.004 | 0.436 | 148.638 | 0.047 | NA | NA |
|  | Myocardial infarction | -0.007 | 0.005 | 0.196 | 144.383 | 0.017 | NA | NA |
|  | Ischemic stroke | -0.001 | 0.006 | 0.852 | 167.563 | 0.003 | NA | NA |
| Positive affect | Atrial fibrillation | -0.003 | 0.005 | 0.601 | 119.804 | 0.788 | NA | NA |
|  | Heart failure | -0.004 | 0.003 | 0.219 | 183.070 | 0.035 | NA | NA |
|  | Myocardial infarction | -0.003 | 0.005 | 0.507 | 212.988 | <0.001 | 11.939 | 0.799 |
|  | Ischemic stroke | -0.002 | 0.005 | 0.658 | 198.925 | 0.003 | NA | NA |

WBS: Well-being spectrum; NA: Not applicable. The MR-Egger intercept showed no evidence for directional pleiotropy. MR-PRESSO global test was significant in many cases but MR-PRESSO distortion test did not identify significantly different estimates following outlier adjustment.

**Table S3.** Cochran’s Q test for the presence of heterogeneity between WBS and constituent traits, and cardiovascular disease outcomes.

| **Exposure** | **Outcome** | **Method** | **Q** | **Q_df** | **Q_pval** |
| --- | --- | --- | --- | --- | --- |
| WBS | Atrial fibrillation | IVW | 210.548 | 182 | 0.072 |
|  |  | MR-Egger | 208.642 | 181 | 0.078 |
|  | Heart failure | IVW | 273.035 | 203 | 0.001 |
|  |  | MR-Egger | 272.911 | 202 | 0.001 |
|  | Myocardial infarction | IVW | 263.151 | 201 | 0.002 |
|  |  | MR-Egger | 262.912 | 200 | 0.002 |
|  | Ischemic stroke | IVW | 282.085 | 202 | <0.001 |
|  |  | MR-Egger | 279.673 | 201 | <0.001 |
| Depressive symptoms | Atrial fibrillation | IVW | 180.754 | 169 | 0.254 |
|  |  | MR-Egger | 180.615 | 168 | 0.240 |
|  | Heart failure | IVW | 223.587 | 189 | 0.043 |
|  |  | MR-Egger | 223.544 | 188 | 0.039 |
|  | Myocardial infarction | IVW | 244.723 | 189 | 0.004 |
|  |  | MR-Egger | 244.501 | 188 | 0.003 |
|  | Ischemic stroke | IVW | 250.528 | 189 | 0.002 |
|  |  | MR-Egger | 250.280 | 188 | 0.002 |
| Neuroticism | Atrial fibrillation | IVW | 205.236 | 179 | 0.087 |
|  |  | MR-Egger | 205.176 | 178 | 0.080 |
|  | Heart failure | IVW | 291.294 | 202 | <0.001 |
|  |  | MR-Egger | 290.076 | 201 | <0.001 |
|  | Myocardial infarction | IVW | 282.400 | 202 | <0.001 |
|  |  | MR-Egger | 281.527 | 201 | <0.001 |
|  | Ischemic stroke | IVW | 262.180 | 202 | 0.003 |
|  |  | MR-Egger | 262.032 | 201 | 0.002 |
| Life satisfaction | Atrial fibrillation | IVW | 117.084 | 106 | 0.217 |
|  |  | MR-Egger | 116.137 | 105 | 0.215 |
|  | Heart failure | IVW | 146.258 | 119 | 0.046 |
|  |  | MR-Egger | 145.504 | 118 | 0.044 |
|  | Myocardial infarction | IVW | 141.865 | 118 | 0.067 |
|  |  | MR-Egger | 139.847 | 117 | 0.074 |
|  | Ischemic stroke | IVW | 164.801 | 119 | 0.003 |
|  |  | MR-Egger | 164.753 | 118 | 0.003 |
| Positive affect | Atrial fibrillation | IVW | 118.041 | 131 | 0.784 |
|  |  | MR-Egger | 117.766 | 130 | 0.771 |
|  | Heart failure | IVW | 180.712 | 148 | 0.035 |
|  |  | MR-Egger | 178.856 | 147 | 0.038 |
|  | Myocardial infarction | IVW | 210.111 | 148 | 0.001 |
|  |  | MR-Egger | 209.482 | 147 | 0.001 |
|  | Ischemic stroke | IVW | 196.093 | 148 | 0.005 |
|  |  | MR-Egger | 195.831 | 147 | 0.004 |

**Table S4.** Univariable Mendelian randomization analyses of cardiovascular risk factors with atrial fibrillation.

| **Outcome** | **Exposure** | **method** | **nSNP** | **OR** | **LCI** | **UCI** | **P** |
| --- | --- | --- | --- | --- | --- | --- | --- |
| Atrial fibrillation | BMI | MR Egger | 91 | 1.058 | 0.979 | 1.145 | 0.159 |
|  |  | Weighted median | 91 | 1.066 | 1.019 | 1.115 | 0.005 |
|  |  | Inverse variance weighted | 91 | 1.078 | 1.043 | 1.114 | 8.59x10^-6^ |
|  | HDL | MR Egger | 65 | 0.861 | 0.615 | 1.204 | 0.385 |
|  |  | Weighted median | 65 | 0.709 | 0.542 | 0.929 | 0.013 |
|  |  | Inverse variance weighted | 65 | 0.867 | 0.685 | 1.097 | 0.235 |
|  | LDL | MR Egger | 48 | 0.877 | 0.732 | 1.051 | 0.163 |
|  |  | Weighted median | 48 | 0.894 | 0.763 | 1.048 | 0.167 |
|  |  | Inverse variance weighted | 48 | 0.912 | 0.810 | 1.027 | 0.129 |
|  | SBP | MR Egger | 104 | 1.018 | 0.967 | 1.072 | 0.501 |
|  |  | Weighted median | 104 | 1.010 | 0.988 | 1.032 | 0.385 |
|  |  | Inverse variance weighted | 104 | 1.022 | 1.005 | 1.039 | 0.011 |
|  | Smoking (never) | MR Egger | 189 | 1.729 | 0.472 | 6.331 | 0.410 |
|  |  | Weighted median | 189 | 1.038 | 0.655 | 1.645 | 0.873 |
|  |  | Inverse variance weighted | 189 | 1.230 | 0.887 | 1.704 | 0.215 |
|  | T2D | MR Egger | 129 | 0.460 | 0.058 | 3.652 | 0.464 |
|  |  | Weighted median | 129 | 0.509 | 0.112 | 2.314 | 0.382 |
|  |  | Inverse variance weighted | 129 | 1.202 | 0.399 | 3.619 | 0.743 |

BMI: body mass index; HDL: High density lipoprotein cholesterol; LDL: Low density lipoprotein cholesterol; SBP: Systolic blood pressure; T2D: Type 2 diabetes; LCI: lower 95% confidence interval; UPI: Upper 95% confidence interval.

**Table S5.** Univariable Mendelian randomization analyses of cardiovascular risk factors with heart failure.

| **Outcome** | **Exposure** | **Method** | **n SNP** | **OR** | **LCI** | **UCI** | **P** |
| --- | --- | --- | --- | --- | --- | --- | --- |
| Heart failure | BMI | MR Egger | 96 | 1.105 | 1.051 | 1.162 | 1.91x10^-4^ |
|  |  | Weighted median | 96 | 1.128 | 1.094 | 1.163 | 1.85x10^-14^ |
|  |  | Inverse variance weighted | 96 | 1.126 | 1.099 | 1.154 | 5.24x10^-22^ |
|  | HDL | MR Egger | 70 | 0.752 | 0.584 | 0.967 | 0.030 |
|  |  | Weighted median | 70 | 0.741 | 0.620 | 0.885 | 0.001 |
|  |  | Inverse variance weighted | 70 | 0.745 | 0.626 | 0.887 | 0.001 |
|  | LDL | MR Egger | 51 | 1.258 | 1.058 | 1.494 | 0.012 |
|  |  | Weighted median | 51 | 1.341 | 1.170 | 1.537 | 2.57x10^-5^ |
|  |  | Inverse variance weighted | 51 | 1.266 | 1.130 | 1.418 | 4.57x10^-5^ |
|  | SBP | MR Egger | 125 | 1.021 | 0.995 | 1.049 | 0.115 |
|  |  | Weighted median | 125 | 1.025 | 1.012 | 1.038 | 1.80x10^-4^ |
|  |  | Inverse variance weighted | 125 | 1.029 | 1.020 | 1.038 | 1.89x10^-10^ |
|  | Smoking (never) | MR Egger | 209 | 0.313 | 0.138 | 0.709 | 0.006 |
|  |  | Weighted median | 209 | 0.557 | 0.427 | 0.727 | 1.64x10^-5^ |
|  |  | Inverse variance weighted | 209 | 0.501 | 0.407 | 0.616 | 6.31x10^-11^ |
|  | T2D | MR Egger | 137 | 0.558 | 0.151 | 2.067 | 0.384 |
|  |  | Weighted median | 137 | 1.270 | 0.501 | 3.221 | 0.615 |
|  |  | Inverse variance weighted | 137 | 3.093 | 1.514 | 6.318 | 0.002 |

BMI: body mass index; HDL: High density lipoprotein cholesterol; LDL: Low density lipoprotein cholesterol; SBP: Systolic blood pressure; T2D: Type 2 diabetes; LCI: lower 95% confidence interval; UPI: Upper 95% confidence interval.

**Table S6.** Univariable Mendelian randomization analyses of cardiovascular risk factors with myocardial infarction.

| **Outcome** | **Exposure** | **Method** | **nSNP** | **OR** | **LCI** | **UCI** | **P** |
| --- | --- | --- | --- | --- | --- | --- | --- |
| Myocardial infarction | BMI | MR Egger | 96 | 1.130 | 1.061 | 1.204 | 2.78x10^-4^ |
|  |  | Weighted median | 96 | 1.093 | 1.054 | 1.132 | 1.10x10^-6^ |
|  |  | Inverse variance weighted | 96 | 1.099 | 1.066 | 1.133 | 1.24x10^-9^ |
|  | HDL | MR Egger | 70 | 0.888 | 0.613 | 1.287 | 0.533 |
|  |  | Weighted median | 70 | 0.692 | 0.563 | 0.850 | 4.65x10^-4^ |
|  |  | Inverse variance weighted | 70 | 0.602 | 0.459 | 0.790 | 2.56x10^-4^ |
|  | LDL | MR Egger | 50 | 2.201 | 1.781 | 2.720 | 2.54x10^-9^ |
|  |  | Weighted median | 50 | 2.024 | 1.771 | 2.313 | 4.23x10^-25^ |
|  |  | Inverse variance weighted | 50 | 2.037 | 1.775 | 2.337 | 4.49x10^-24^ |
|  | SBP | MR Egger | 125 | 1.049 | 1.012 | 1.088 | 0.011 |
|  |  | Weighted median | 125 | 1.038 | 1.023 | 1.054 | 6.85x10^-7^ |
|  |  | Inverse variance weighted | 125 | 1.045 | 1.033 | 1.058 | 1.00x10^-12^ |
|  | Smoking (never) | MR Egger | 207 | 0.783 | 0.294 | 2.084 | 0.625 |
|  |  | Weighted median | 207 | 0.527 | 0.387 | 0.716 | 4.38x10^-5^ |
|  |  | Inverse variance weighted | 207 | 0.522 | 0.408 | 0.669 | 2.59x10^-7^ |
|  | T2D | MR Egger | 137 | 3.034 | 0.592 | 15.532 | 0.185 |
|  |  | Weighted median | 137 | 10.116 | 3.168 | 32.297 | 9-34x10^-5^ |
|  |  | Inverse variance weighted | 137 | 24.208 | 9.925 | 59.047 | 2.47x10^-12^ |

BMI: body mass index; HDL: High density lipoprotein cholesterol; LDL: Low density lipoprotein cholesterol; SBP: Systolic blood pressure; T2D: Type 2 diabetes; LCI: lower 95% confidence interval; UPI: Upper 95% confidence interval.

**Table S7.** Univariable Mendelian randomization analyses of cardiovascular risk factors with ischemic stroke.

| **Outcome** | **Exposure** | **Method** | **nSNP** | **OR** | **LCI** | **UCI** | **P** |
| --- | --- | --- | --- | --- | --- | --- | --- |
| Ischemic stroke | BMI | MR Egger | 96 | 1.025 | 0.971 | 1.082 | 0.378 |
|  |  | Weighted median | 96 | 1.025 | 0.988 | 1.064 | 0.183 |
|  |  | Inverse variance weighted | 96 | 1.035 | 1.008 | 1.062 | 0.010 |
|  | HDL | MR Egger | 70 | 1.251 | 1.014 | 1.544 | 0.041 |
|  |  | Weighted median | 70 | 1.050 | 0.847 | 1.302 | 0.658 |
|  |  | Inverse variance weighted | 70 | 0.887 | 0.757 | 1.039 | 0.136 |
|  | LDL | MR Egger | 50 | 1.148 | 0.931 | 1.414 | 0.202 |
|  |  | Weighted median | 50 | 1.036 | 0.905 | 1.186 | 0.612 |
|  |  | Inverse variance weighted | 50 | 1.081 | 0.941 | 1.240 | 0.270 |
|  | SBP | MR Egger | 125 | 1.035 | 0.997 | 1.075 | 0.074 |
|  |  | Weighted median | 125 | 1.039 | 1.023 | 1.056 | 1.47x10^-6^ |
|  |  | Inverse variance weighted | 125 | 1.048 | 1.035 | 1.062 | 3.98x10^-13^ |
|  | Smoking (never) | MR Egger | 209 | 0.400 | 0.137 | 1.171 | 0.096 |
|  |  | Weighted median | 209 | 0.701 | 0.501 | 0.981 | 0.038 |
|  |  | Inverse variance weighted | 209 | 0.666 | 0.508 | 0.874 | 0.003 |
|  | T2D | MR Egger | 137 | 1.583 | 0.417 | 6.007 | 0.501 |
|  |  | Weighted median | 137 | 7.297 | 2.389 | 22.289 | 4.86x10^-4^ |
|  |  | Inverse variance weighted | 137 | 7.935 | 3.877 | 16.239 | 1.44x10^-8^ |

BMI: body mass index; HDL: High density lipoprotein cholesterol; LDL: Low density lipoprotein cholesterol; SBP: Systolic blood pressure; T2D: Type 2 diabetes; LCI: lower 95% confidence interval; UPI: Upper 95% confidence interval.

**Table S8.** Reverse Mendelian randomization analyses of myocardial infarction (as exposure) with well-being spectrum and four well-being traits (as outcome).

| **Exposure** | **Outcome** | **Method** | **nSNP** | **Beta** | **LCI** | **UCI** | **P** |
| --- | --- | --- | --- | --- | --- | --- | --- |
| Myocardial infarction | Well-being spectrum | Inverse variance weighted | 34 | -0.001 | -0.008 | 0.005 | 0.679 |
|  |  | Weighted median | 34 | -0.004 | -0.011 | 0.003 | 0.228 |
|  |  | MR Egger | 34 | 0.003 | -0.013 | 0.018 | 0.738 |
|  | Positive affect | Inverse variance weighted | 34 | -0.001 | -0.007 | 0.006 | 0.871 |
|  |  | Weighted median | 34 | -0.004 | -0.012 | 0.005 | 0.411 |
|  |  | MR Egger | 34 | 0.004 | -0.012 | 0.020 | 0.612 |
|  | Life satisfaction | Inverse variance weighted | 34 | -0.002 | -0.010 | 0.006 | 0.650 |
|  |  | Weighted median | 34 | -0.007 | -0.016 | 0.002 | 0.119 |
|  |  | MR Egger | 34 | 0.001 | -0.019 | 0.020 | 0.963 |
|  | Depressive symptoms | Inverse variance weighted | 34 | 0.002 | -0.005 | 0.008 | 0.649 |
|  |  | Weighted median | 34 | 0.005 | -0.002 | 0.012 | 0.138 |
|  |  | MR Egger | 34 | -0.003 | -0.019 | 0.012 | 0.658 |
|  | Neuroticism | Inverse variance weighted | 34 | 0.002 | -0.008 | 0.013 | 0.670 |
|  |  | Weighted median | 34 | 0.008 | -0.002 | 0.019 | 0.111 |
|  |  | MR Egger | 34 | -0.006 | -0.030 | 0.018 | 0.648 |

nSNP: number of single nucleotide polymorphisms; LCI: Lower 95% confidence interval; UCI: Upper 95% confidence interval.
